# Conflicting findings on the effectiveness of hydrogen therapy for ameliorating vascular leakage in a 5-day post hypoxic-ischemic survival piglet model

**DOI:** 10.1038/s41598-023-37577-0

**Published:** 2023-06-28

**Authors:** Yinmon Htun, Shinji Nakamura, Yasuhiro Nakao, Tsutomu Mitsuie, Kenichi Ohta, Makoto Arioka, Takayuki Yokota, Eri Inoue, Kota Inoue, Toi Tsuchiya, Kosuke Koyano, Yukihiko Konishi, Takanori Miki, Masaki Ueno, Takashi Kusaka

**Affiliations:** 1 Department of Pediatrics, Faculty of Medicine, Kagawa University, 1750-1 Mikicho, Kitagun, Kagawa 761-0793 Japan; 2 Medical Engineering Equipment Management Center, Kagawa University Hospital, Kagawa University, 1750-1 Mikicho, Kitagun, Kagawa 761-0793 Japan; 3 Department of Anatomy and Neurobiology, Faculty of Medicine, Kagawa University, 1750-1 Mikicho, Kitagun, Kagawa 761-0793 Japan; 4 Maternal and Perinatal Center, Kagawa University Hospital, Kagawa University, 1750-1 Mikicho, Kitagun, Kagawa 761-0793 Japan; 5 Department of Pathology and Host Defense, Faculty of Medicine, Kagawa University, 1750-1 Mikicho, Kitagun, Kagawa 761-0793 Japan

**Keywords:** Neuroscience, Diseases, Medical research

## Abstract

Neonatal hypoxic-ischemic encephalopathy (HIE) is a major cause of morbidity and mortality in newborns in both high- and low-income countries. The important determinants of its pathophysiology are neural cells and vascular components. In neonatal HIE, increased vascular permeability due to damage to the blood–brain barrier is associated with seizures and poor outcomes in both translational and clinical studies. In our previous studies, hydrogen gas (H_2_) improved the neurological outcome of HIE and ameliorated the cell death. In this study, we used albumin immunohistochemistry to assess if H_2_ inhalation effectively reduced the cerebral vascular leakage. Of 33 piglets subjected to a hypoxic-ischemic insult, 26 piglets were ultimately analyzed. After the insult, the piglets were grouped into normothermia (NT), H_2_ ventilation (H_2_), therapeutic hypothermia (TH), and H_2_ combined with TH (H_2_-TH) groups. The ratio of albumin stained to unstained areas was analyzed and found to be lower in the H_2_ group than in the other groups, although the difference was not statistically significant. In this study, H_2_ therapy did not significantly improve albumin leakage despite the histological images suggesting signs of improvement. Further investigations are warranted to study the efficacy of H_2_ gas for vascular leakage in neonatal HIE.

## Introduction

Hypoxic-ischemic encephalopathy (HIE) is a major cause of neonatal death and neurological disability. Annually, more than 1 million neonates die of HIE-related complications, and its incidence ranges from 1 to 8/1000 live births in high-income countries and to as high as 26/1000 live births in low-/middle-income countries^1,2^. Although the standard treatment is therapeutic hypothermia (TH), just 1 in 7–8 treated neonates benefits^3^. Regarding neonatal HIE findings, watershed injury is associated with cognitive impairments in neonates with moderate-to-severe HIE while subcortical structural damage results in motor dysfunctions^4–6^. In a piglet model, neuroprotection can be region-specific, with the posterior putamen not protected by TH^7^. Thus, to further improve outcomes, a new therapeutic strategy for HIE is necessary.

HIE is an evolving process that involves a series of biochemical cascades that lead to neurovascular injury and cell death over days to even years^8^. In HIE, interruption of the cerebral blood flow, oxygen depletion, energy failure, and reoxygenation contribute to the release of free radicals^9^. Under hypoxic conditions, due to the release of free radicals and inflammatory cytokines and the enhanced production of nitric oxide (NO) and vascular endothelial growth factor (VEGF), the barriers of the brain such as the blood–brain barrier (BBB) become disrupted. The BBB is a permeability barrier comprising neurovascular units that regulate the exchange of materials between the systemic circulation and brain parenchyma. After a hypoxic-ischemic (HI) insult, angiogenesis and permeability changes undermine the integrity of the BBB, making it vulnerable to edema formation and allowing the leakage of vascular components such as albumin^10–12^. BBB damage in a translational piglet model is associated with seizures, which result in poor outcomes and greater histological damage^13^. In the same study, BBB disruption was associated with leakage of IgG proteins into the brain and their increased uptake by neurons and with upregulation of inflammatory cytokines and elevations in their gene expression. In studies of HIE neonates, an increased cerebrospinal fluid/plasma albumin ratio indicates aggravated vascular leakage and underlying free radical injury^14,15^. Therefore, a novel therapeutic strategy focusing on oxidative stress-related BBB damage may be crucial for better outcomes in HIE.

**Figure 1:**
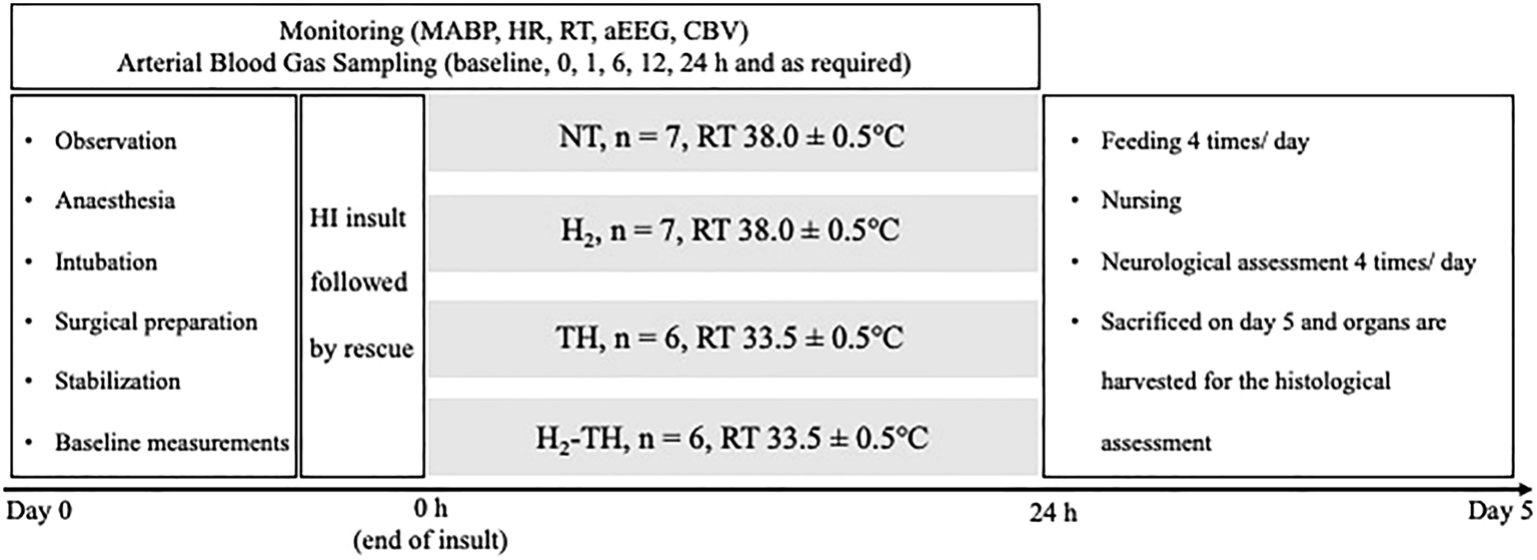
Timeline of the experiment. On day 0, an HI insult was performed after surgical preparation and stabilization. The HI insult was followed by rescue with 100% O_2_. After the rescue, the piglets were randomized into NT, H_2_, TH, and H_2_-TH groups and the treatment was given accordingly for 24 h. In the TH and H_2_-TH groups, rewarming was performed. From day 1 to day 5, the piglets were allowed to recover, nursed, and fed. Neurological function was observed every 6 h from day 1 to day 5 after the insult. On day 5, the piglets were euthanized and their brains were harvested for histological analysis.

Molecular hydrogen (H_2_) is one potential agent for alleviating the vascular leakage and improving BBB integrity. H_2_ is a free radical scavenger that is especially selective for hydroxyl radical (·OH), which has no known physiological role and indiscriminately reacts with cell membrane proteins and lipids^16^. In translational studies related to ischemia–reperfusion brain injury, including HIE, H_2_ ameliorated the BBB disruption and the subsequent development of edematous changes^17–19^. Our previous study, conducted using an original translational protocol (Fig. 1), showed that TH combined with H_2_ improved motor function in a 5-day post HI piglet model in which the HI insult was controlled using cerebral blood volume (CBV), amplitude-integrated EEG (aEEG), and physiological parameters (mean arterial blood pressure [MABP] and heart rate [HR])^20,21^. Also, significant histological improvements were seen in part of the cortex in the combined therapy group in our previous study. Therefore, based on its anti-oxidative, anti-inflammatory, and anti-apoptotic mechanisms^22^, we hypothesized that H_2_ therapy might effectively ameliorate the vascular leakage.

In studies of neonatal HIE, piglet models serve as a major preclinical step toward the clinical application of novel therapeutic strategies. Piglets are similar to human neonates in various ways, including the timing of the brain growth spurt, myelination patterns, gross neuroanatomical features (a gyrencephalic cerebral cortex, similar white matter pathways, and lobe structures similar to Brodmann cortical areas), cerebral metabolic rates, a brain weight that is about 35–40% of that of the adult, and similarities at the genetic level^23–25^. In addition, their body weights and sizes are similar (1.5–2.5 kg), allowing the piglet models to simulate neonatal intensive care unit conditions. Accordingly, piglet models permit similar clinical management and physiological and neurological monitoring.

None of the piglet models developed at various institutions are identical, with each having its own HI protocol, modalities for controlling the HI insult, duration of survival, therapeutic strategy, and primary outcome biomarkers. Although there are many models, we will briefly mention the two main types of piglet models. The first type maintains mechanical ventilation from the start to the end of the experiment (non-recovery models, 24–72-h continuous monitoring). These models continuously monitor the changes in cerebral activities and hemodynamics and have allowed us to better understand the pathophysiology and phases of neonatal HIE and established HI insult protocols and to tailor the treatment strategy to the phase^26–30^. The HI insult protocols in such models involve invasive surgical procedures such as tracheostomy and carotid artery ligation^24^. The second type is a recovery model that allows the piglet to wean from mechanical ventilation, which enables neurological assessments. These models permit observation of neurological outcomes in a relatively long-term manner. Moreover, the above-mentioned invasive procedures are not suitable. The features of survival models are oral intubation and a lowering of the FiO_2_ and MABP (hypoxic and ischemic components, respectively), as well as feeding and nursing care after weaning from mechanical ventilation^20,30,31^. Both models have helped us to develop HI insult protocols, monitoring modalities, and treatment protocols.

Regarding H_2_ therapy in piglet models, a 24-h non-recovery model provided valuable evidence that H_2_ therapy itself has neuroprotective potential in neonatal HIE. In those studies, the HI insult was induced by 20 min of asphyxia (reduction of the FiO_2_ to 6%, respiration rate 15 L/min). Four-hour ventilation with 2.1% H_2_ restored brain activity and reduced the histological damage at 24 h of survival and inhibited the upregulation of brain cyclooxygenase-2 expression (an enzyme with a role in neuroinflammation)^27,32–34^. However, when combined with TH (given 2–36 h after the insult), H_2_ did not augment the neuroprotection of TH in a 48-h non-recovery model^35^. On the other hand, our survival model is suitable for assessing neurological outcomes for 5 days after the insult. The HI insult protocol for this model is described in the Methods section (Fig. 1). A unique feature of this recovery model is that absolute values of CBV combined with brain activity are monitored to produce a recovery model with sustained brain injuries^21^. In our previous study, H_2_ ventilation combined with TH improved neurological outcomes and moderately protected part of the cortex^20^.

By using our 5-day post HI insult piglet model, we investigated the effectiveness of H_2_ in terms of vascular leakage in this study. The study is reported in accordance with Animal Research: Reporting of In Vivo Experiments.

## Results

### Duration of low-amplitude-integrated EEG

There were no significant differences in the mean total duration (standard error of the mean [SEM]) of low-amplitude-integrated EEG (LAEEG) among the four groups: normothermia (NT), 47.4 (1.4) min; H_2_, 41.6 (2.7) min; TH, 48.3 (2.7) min; and H_2_-TH, 43.2 (3.9) min.

### Physiological and biochemical data

There were no significant differences among the four groups in HR, MABP, or rectal temperature (RT) at baseline (Table 1). The NT, H_2_, and H_2_-TH groups showed a significant reduction in HR at 0 h (end of insult), whereas the TH group showed a nonsignificant reduction in HR compared with baseline. HR had returned to baseline values by 1 h after the insult in all four groups. At 6 and 12 h after the insult, HR was significantly higher in the NT group than in the H_2_ group. At 6, 12, and 24 h after the insult, the H_2_-TH group had a significantly reduced HR compared with baseline.

**Table 1:** Physiological parameters at baseline, 0 h (end of insult), 1 h, 6 h, 12 h, and 24 h after the insult.

Parameters	Baseline	0 h	1 h	6 h	12 h	24 h
HR (bpm)						
NT	212.7 ± 22.9	150.7 ± 17.4***	223.1 ± 21.5	228.1 ± 19.6^§^	235.1 ± 16.4^§^	195.1 ± 36.7
H_2_	179.7 ± 14.9	140.4 ± 32.7*	207.9 ± 15.3	230.1 ± 28.7**^,§^	217.1 ± 17.1^§^	183.4 ± 31.8
TH	211.2 ± 47.0	175.2 ± 48.2	207.0 ± 38.9	213.3 ± 8.1	235.1 ± 16.4	195.1 ± 36.7
H_2_-TH	215.0 ± 11.1	166.8 ± 17.1**	239.7 ± 18.2	179.0 ± 19.9*	172.7 ± 30.0*	177.3 ± 23.1*
MABP (mmHg)						
NT	79.6 ± 6.5	48.6 ± 12.7****	64.0 ± 6.7*	72.0 ± 11.3	67.1 ± 12.6	61.0 ± 9.8*
H_2_	72.7 ± 6.3	45.6 ± 11.1***	66.4 ± 8.3	69.4 ± 6.7	66.9 ± 9.2	55.4 ± 3.8**
TH	75.3 ± 15.9	50.8 ± 10.7**	76.2 ± 6.7	66.8 ± 3.5	68.3 ± 8.2	59.7 ± 7.0
H_2_-TH	76.2 ± 4.1	49.4 ± 15.3***	68.3 ± 2.9	71.7 ± 8.5	64.5 ± 7.8	63.5 ± 3.6
RT (°C)						
NT	37.5 ± 0.7	37.6 ± 0.5	38.2 ± 0.6^#,§^	38.4 ± 0.4*^,#,§^	38.0 ± 0.4^#,§^	38.3 ± 0.6^#,§^
H_2_	38.2 ± 0.3	38.1 ± 0.3^#^	38.2 ± 0.4^#,§^	38.3 ± 0.3^#,§^	38.4 ± 0.5^#,§^	38.8 ± 0.2*^,#,§^
TH	36.8 ± 0.8	36.8 ± 0.8	33.9 ± 0.8****^,§^	33.9 ± 0.3****	34.0 ± 0.4****^,§^	34.1 ± 0.4****^,§^
H_2_-TH	38.1 ± 0.8	37.6 ± 1.0	36.4 ± 1.1	33.4 ± 0.6****	32.9 ± 1.0****	35.3 ± 1.6**

Values are expressed as the mean ± SD. *NT* normothermia, *TH* therapeutic hypothermia, *H*_*2*_ hydrogen ventilation, *TH-H*_*2*_ therapeutic hypothermia with hydrogen ventilation. *p < 0.05, **p < 0.01, ***p < 0.001, ****p < 0.0001 versus baseline; ^#^p < 0.05 versus TH; ^§^p < 0.05 versus H_2_-TH; ^†^p < 0.05 versus H_2_.

There was a significant decline in MABP at the end of the insult compared with baseline in all groups. In the NT group, MABP at 1 and 24 h after the insult was significantly reduced compared with baseline. The piglets in the H_2_ group had a significantly lower MABP at 24 h after the insult compared with baseline. MABP values were not significantly different among any of the groups at any time point.

The baseline values for RT were similar in all groups, although the RT was slightly lower in the TH group. In the NT group, the RT was higher than at baseline at 6 h after the insult. The RT of the NT group was significantly higher than that of the TH and H_2_-TH groups at 1, 6, 12, and 24 h after the insult. In the TH group, the RT was significantly lower than at baseline and at the end of insult at 1, 6, 12, and 24 h after the insult. At 24 h after the insult, the TH group had a lower temperature than the NT and H_2_ groups. In the H_2_ group, the RT was largely constant throughout the experiment, except at 24 h. At 0, 1, 6, 12, and 24 h, the RT of the H_2_ group was higher than that of the TH and H_2_-TH groups. In the H_2_-TH group, the RT was lower at 6, 12, and 24 h than at baseline. From 1 h after the insult, the RT of the H_2_-TH group was lower than that of the NT and H_2_ groups.

Biochemical parameters such as pO_2_, pCO_2_, pH, base excess, lactate, and glucose at baseline showed no significant differences among the four groups (Table 2). pH, pO_2_, and base excess were significantly reduced at 0 h (end of insult) and blood lactate was significantly higher at 0 h in all groups compared with their respective baseline values. The huge drop in the base excess paralleled the significant rise in blood lactate levels at 0 and 1 h after the insult, and it gradually returned to normal values. The pH was lowest at the end of the insult and then gradually rose. The glucose level was higher in the TH group than in the other groups, albeit not significantly. The pH was lowest at the end of the insult and gradually increased thereafter. pCO_2_ was maintained at a constant value for 24 h after the insult in all groups.

**Table 2:** Arterial blood gas data at baseline, 0 h (end of insult), 1 h, 6 h, 12 h, and 24 h after the insult.

Parameters	Baseline	0 h	1 h	6 h	12 h	24 h
pH						
NT	7.42 ± 0.05	6.81 ± 0.06****^,§,†^	7.28 ± 0.05***	7.46 ± 0.04	7.47 ± 0.04	7.49 ± 0.05^§^
H_2_	7.44 ± 0.05	6.99 ± 0.08****^,#^	7.35 ± 0.08*	7.49 ± 0.03	7.51 ± 0.04	7.49 ± 0.05^§^
TH	7.44 ± 0.10	6.79 ± 0.09****	7.21 ± 0.09***^,§,†^	7.45 ± 0.05	7.43 ± 0.05	7.42 ± 0.04
H_2_-TH	7.43 ± 0.04	6.92 ± 0.10****^,#^	7.35 ± 0.09	7.41 ± 0.04	7.39 ± 0.05^†^	7.37 ± 0.03
pCO_2_ (mmHg)						
NT	45.1 ± 4.0	29.9 ± 9.0**	39.5 ± 6.7	45.6 ± 7.7	43.7 ± 6.9	38.5 ± 3.7
H_2_	44.0 ± 3.8	36.4 ± 12.4	39.4 ± 2.2	40.8 ± 1.8	38.1 ± 3.9	42.0 ± 12.1
TH	42.5 ± 12.8	42.0 ± 12.8^#^	43.6 ± 6.9	40.9 ± 7.5	41.3 ± 7.3	37.0 ± 6.0
H_2_-TH	42.0 ± 4.0	35.5 ± 4.3	37.4 ± 2.9	43.1 ± 4.4	43.8 ± 2.0	43.6 ± 7.5
pO_2_ (mmHg)						
NT	97.3 ± 23.7	17.8 ± 6.5****	91.6 ± 27.9	86.6 ± 9.0	84.2 ± 14.2	91.0 ± 13.5
H_2_	100.9 ± 10.8	19.5 ± 6.0****	107.4 ± 20.3	91.6 ± 17.8	108.8 ± 24.0	117.3 ± 21.6^#^
TH	94.8 ± 11.9	21.0 ± 6.9****	117.7 ± 24.4	79.4 ± 24.5	82.9 ± 24.4	73.7 ± 17.9
H_2_-TH	105.1 ± 3.8	19.9 ± 7.1****	116.3 ± 16.0	104.8 ± 18.8	96.1 ± 13.3	112.0 ± 29.6^#^
Base excess (mmol/L)						
NT	5.2 ± 2.3	− 28.2 ± 2.6****^,§,†^	− 7.6 ± 2.1****	7.7 ± 1.7^§^	6.8 ± 2.9^§^	6.6 ± 2.1^#,§^
H_2_	5.7 ± 2.5	− 21.1 ± 3.4****^,#^	− 3.1 ± 5.0***^,#^	7.3 ± 2.4	6.6 ± 2.8	5.0 ± 3.1^#,§^
TH	3.2 ± 2.1	− 27.4 ± 3.5****^,†^	− 10.0 ± 4.7****^,§^	3.7 ± 2.9	2.9 ± 3.2	0.1 ± 3.1
H_2_-TH	3.5 ± 1.8	− 22.5 ± 3.2****	− 4.2 ± 4.2**^,^	2.5 ± 3.7	1.9 ± 3.1	− 0.5 ± 3.6
Lactate (mmol/L)						
NT	1.9 ± 0.6	26.0 ± 2.6****^,§,†^	14.4 ± 3.1****^,§,†^	3.2 ± 1.1	4.2 ± 1.0	3.6 ± 1.3
H_2_	1.7 ± 0.5	18.9 ± 3.4****^,#^	10.9 ± 2.7****	3.1 ± 0.5	3.6 ± 1.0	5.2 ± 2.1*
TH	2.4 ± 1.1	23.9 ± 3.3****	13.4 ± 3.7****	4.2 ± 1.7	4.7 ± 1.8	5.1 ± 1.3
H_2_-TH	1.9 ± 0.3	19.9 ± 1.8****^,#^	10.6 ± 2.1****	3.6 ± 0.8	4.0 ± 0.7	5.3 ± 2.7**
Glucose (mmol/L)						
NT	7.9 ± 1.1	13.3 ± 4.9*^,†^	13.7 ± 3.0*	10.3 ± 3.2	11.1 ± 4.2	6.4 ± 2.4
H_2_	7.1 ± 1.2	7.8 ± 2.9	10.8 ± 3.1**	8.3 ± 1.7	7.6 ± 1.5	4.8 ± 0.8
TH	8.5 ± 2.6	12.3 ± 6.6	13.6 ± 4.3	12.5 ± 4.0	13.1 ± 4.4^†^	11.0 ± 3.0^†^
H_2_-TH	8.1 ± 1.2	12.3 ± 4.7	10.9 ± 3.5	11.2 ± 1.8	12.7 ± 1.5*^,†^	9.1 ± 3.5

Values are expressed as the mean ± SD. *NT* normothermia, *TH* therapeutic hypothermia, *H*_*2*_ hydrogen ventilation, *TH-H*_*2*_ therapeutic hypothermia with hydrogen ventilation. *p < 0.05, **p < 0.01, ***p < 0.001, ****p < 0.0001 versus baseline; ^#^p < 0.05 versus TH; ^§^p < 0.05 versus H_2_-TH; ^†^p < 0.05 versus H_2_.

### Albumin immunohistochemistry

**Figure 2:**
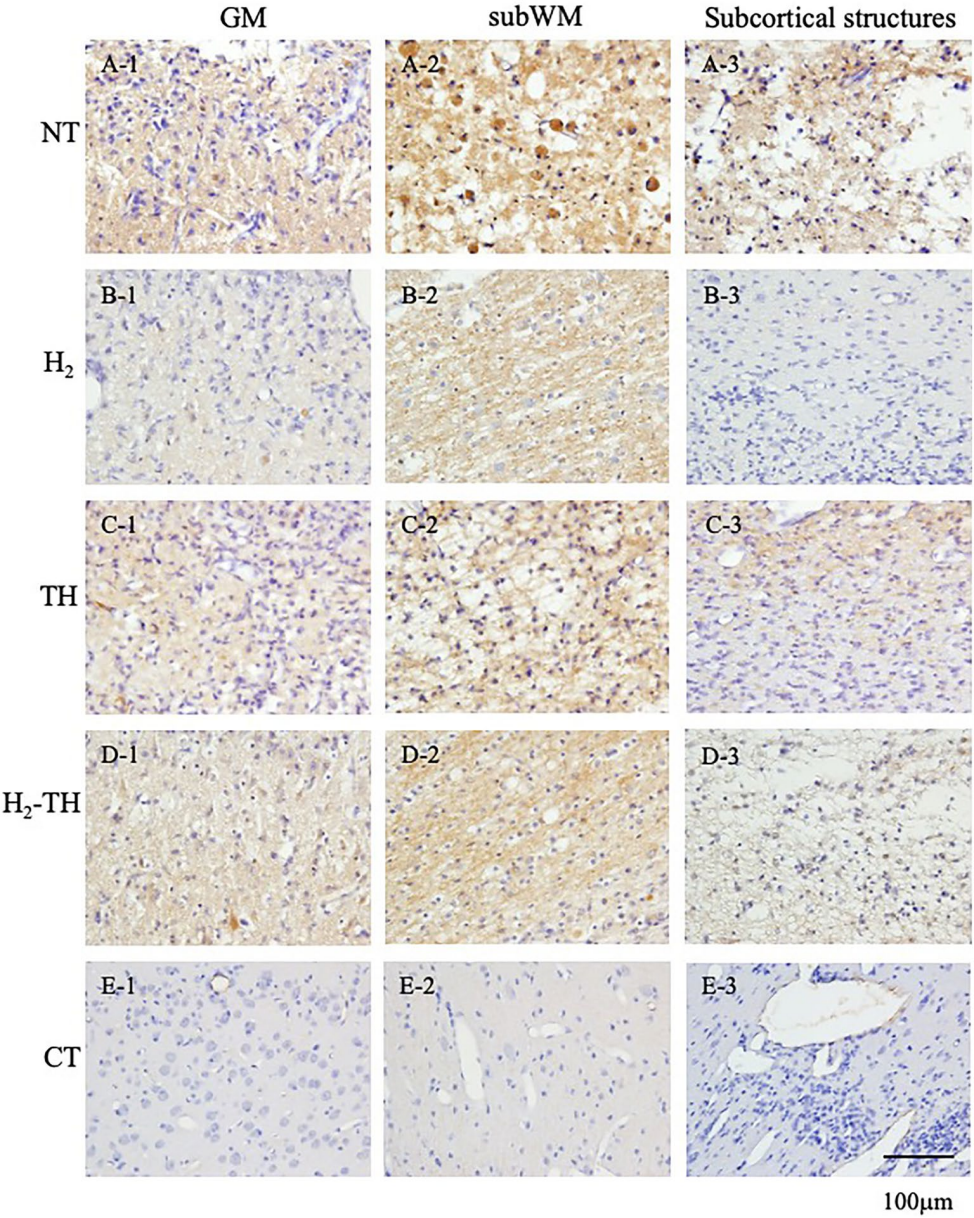
Representative images of albumin immunostaining of the GM (**A1**, **B1**, **C1**, **D1**, and **E1**), subWM (**A2**, **B2**, **C2**, **D2**, and **E2**), and subcortical structures, comprising the periventricular WM and deep nuclear structures (**A3**, **B3**, **C3**, **D3**, and **E3**), in the NT (**A**), H_2_ (**B**), TH (**C**), and H_2_-TH (**D**) groups and the control (**E**). Scale bar, 100 μm.

**Figure 3:**
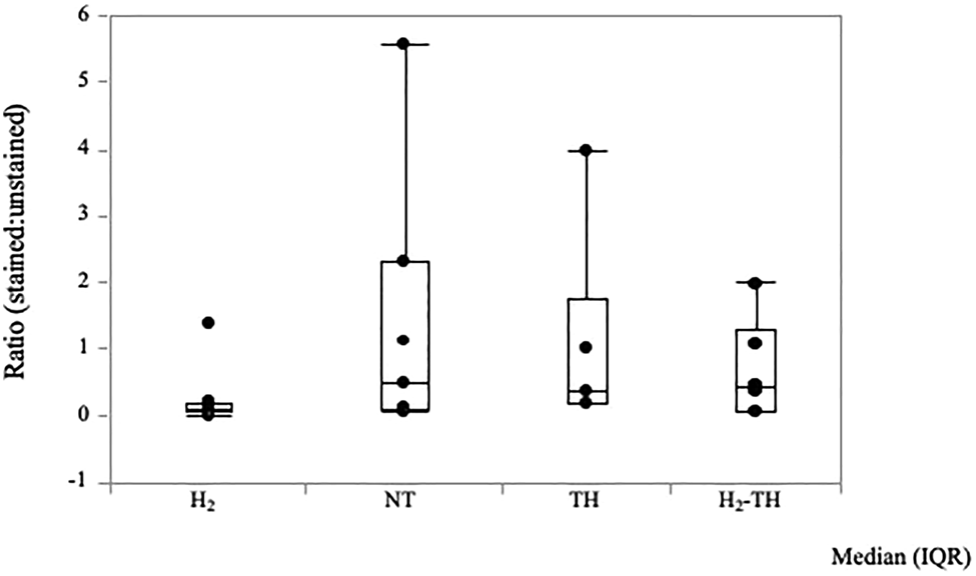
Immunohistochemistry results for the ratio of albumin stained to unstained areas. Steel–Dwass test for multiple comparison showed no significant differences among the four groups. The H_2_ group had the lowest ratio of albumin stained to unstained areas, followed by H_2_-TH, TH, and NT, in that order. The values are expressed as the median (interquartile range).

In this study, the ratio of albumin stained to unstained areas was analyzed and compared among the four groups. Representative images of albumin immunostaining in the GM, subWM, and subcortical structures in the NT, H_2_, TH, and H_2_-TH groups are shown in Fig. 2. Immunohistochemical images of albumin in the GM, subWM, and subcortical structures in all piglets of the four groups are shown and explained in Supplementary Figs. 1–3, respectively. Vascular permeability in the GM in all groups is shown in Supplementary Fig. 4. For the albumin stained to unstained area ratios, median values (interquartile range) were 0.1 (0.17) in the H_2_ group, 0.5 (2.22) in the NT group, 0.38 (1.56) in the TH group, and 0.43 (1.23) in the H_2_-TH group. The *p* values were 0.38 for H_2_ vs NT, 0.21 for H_2_ vs TH, and 0.53 for H_2_ vs H_2_-TH, respectively, showing no statistically significant differences among the groups (Fig. 3).

**Figure 4:**
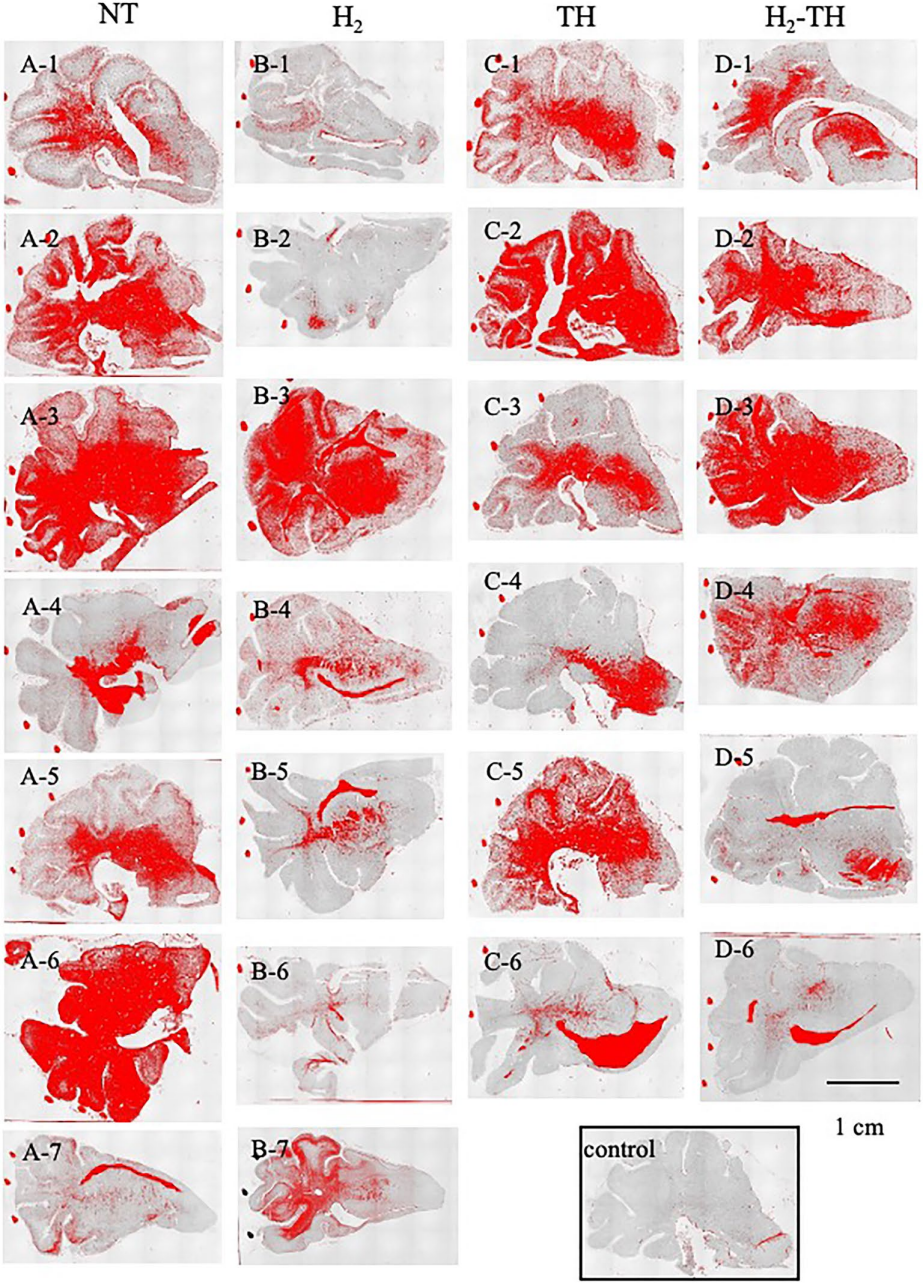
Representative images of albumin-immunoreactive areas under low-power magnification (× 2). (**A1**–**A7**) represent NT, (**B1**–**B7**) represent H_2_, (**C1**–**C6**) represent TH, (**D1**–**D6**) represent H_2_-TH, and (**E**) represents Control. The red dots and red areas in the ventricles were excluded from the analysis. Scale bar, 1 cm.

Albumin immunoreactivity under a low-power magnification is presented in Fig. 4. The color-binarized images show reactive areas in red. The red solid dots outside of the brain in all slides and the red areas in the ventricles (A7, B4, B5, C6, D5, and D6) were excluded from the analysis. In the NT group, the entire section was immunoreactive in almost all of the slides and the leakage was more apparent in the central regions than in the periphery. A similar pattern of immunoreactivity was observed in the remaining groups.

### Neurological scores

**Figure 5:**
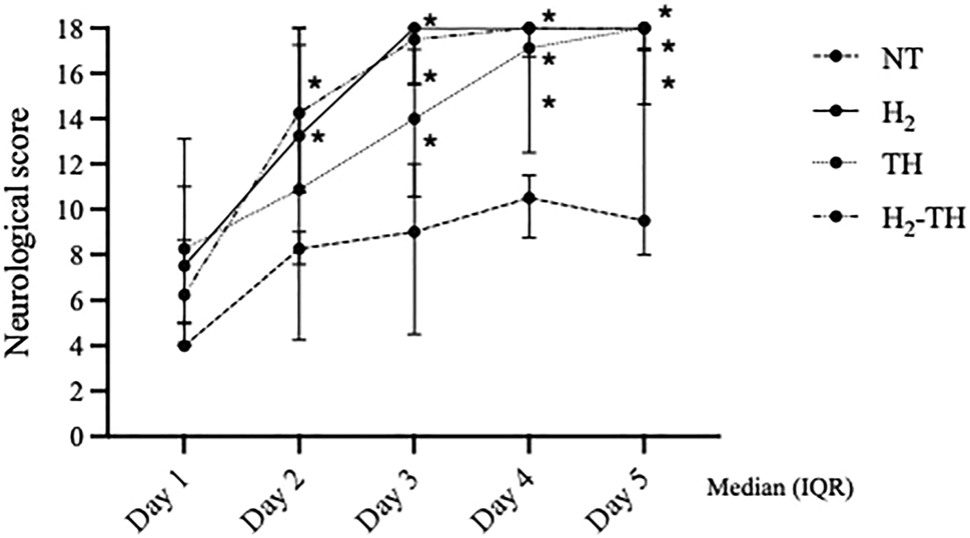
Neurological scores from day 1 to day 5 after HI insult. From day 2 to day 5, the NS was significantly higher in the H_2_ and TH-H_2_ groups compared with the NT group. From day 3, the NS of the TH group was also significantly higher than that of the NT group (p < 0.05 versus NT). All piglets in the H_2_ group reached the full score of 18 at day 5 after the insult (long dashed line, NT; solid line, H_2_; dotted line, TH; and dot-dash line, H_2_-TH). The values are expressed as the median (interquartile range).

The neurological score (NS) was assessed every 6 h from day 1 to day 5 after the HI insult. The NSs of all four groups showed a progressively increasing trend from day 1 to day 5. The NT group had the lowest score of all groups. At day 5 after the insult, all piglets in the H_2_ group had achieved the maximum possible NS, which is 18.0, unlike the other groups. On day 2 after the insult, the NSs of the H_2_ and H_2_-TH groups were significantly higher than that of the NT group (p < 0.05). From day 3 to day 5, the NSs of the H_2_, H_2_-TH, and TH groups showed a significant increase compared with the NT group. However, there were no significance differences among the H_2_, H_2_-TH, and TH groups (Fig. 5).

### Cerebral hemodynamics and oxygenation

**Figure 6:**
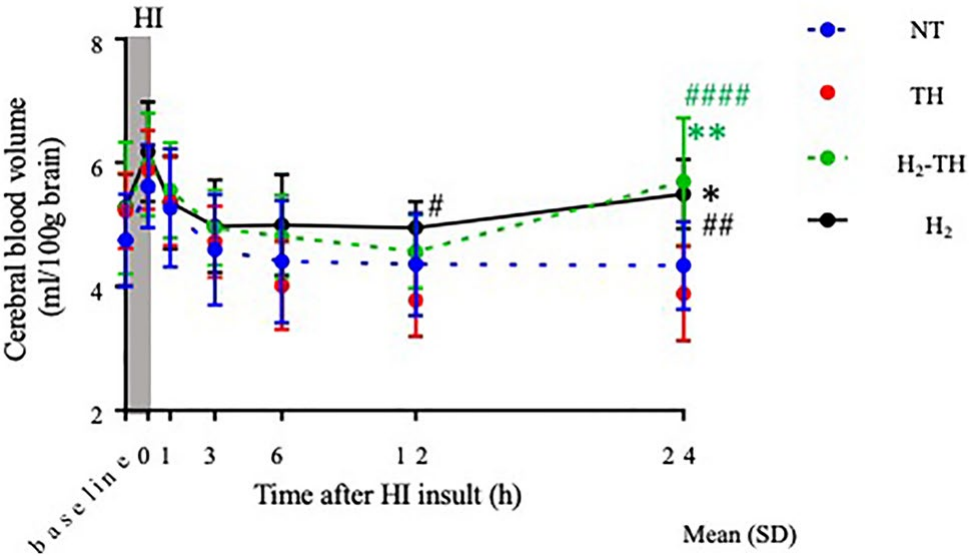
Cerebral blood volume (CBV) values within 24 h after insult. At the end of the insult, CBV values increased in all four groups, followed by a decreasing trend for the NT, TH, and H_2_-TH until 12 h after insult. However, the CBV values of the H_2_-TH group rose from 12 to 24 h after insult. CBV in the H_2_ group showed a relatively stable trend and the values increased between 12 and 24 h. At 24 h after insult, the CBV values of both the H_2_-TH and H_2_ groups were significantly higher than those in the NT and TH groups. The values are expressed as the mean (SD).

There were no statistically significant differences in cerebral hemodynamics compared with baseline in any of the four groups. At the end of the insult, CBV in all four groups increased and then gradually returned to around the respective baseline values 1 h later. Thereafter, the values continued to decrease until 6 h after insult with the TH group showing the lowest CBV. At 12 h after insult, the CBV value was significantly higher in the H_2_ group than in the TH group. At 24 h after insult, CBV in the TH-H_2_ group was the highest among the four groups, with significantly higher values compared with the NT and TH groups. CBV was also significantly higher in the H_2_ group than in the NT and TH groups at 24 h after insult (Fig. 6).

**Figure 7:**
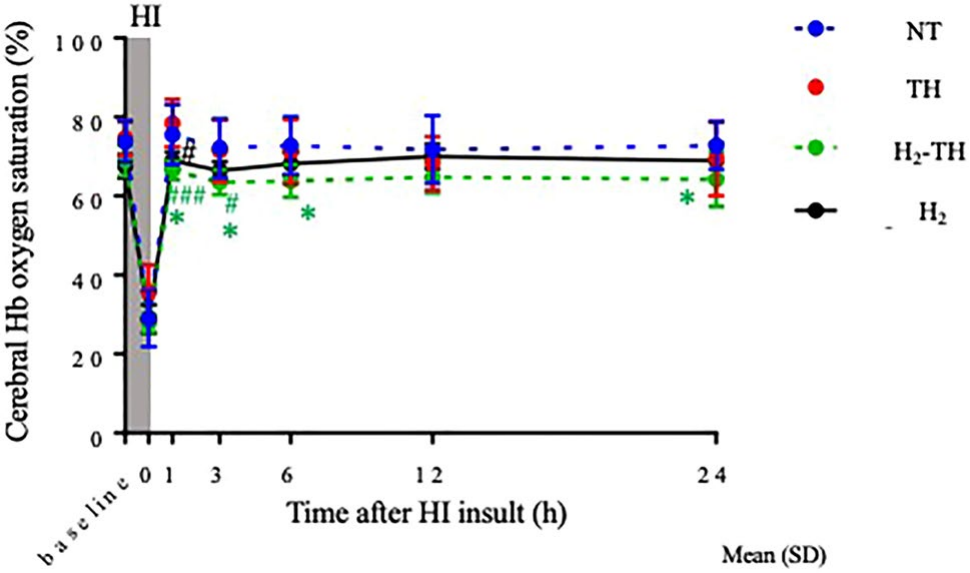
Cerebral hemoglobin oxygenation (ScO_2_) values. ScO_2_ values in all four groups dropped at the end of the insult and then gradually increased to return to around the baseline values. ScO_2_ was the lowest in the H_2_-TH group and the highest in the NT group. The values are expressed as the mean (SD).

No statistically significant differences were noted in cerebral hemoglobin oxygen saturation (ScO_2_) at baseline. ScO_2_ values dropped at the end of the insult compared with baseline in all four groups. At 1, 3, 6, and 24 h after insult, ScO_2_ values were significantly lower in the TH-H_2_ group compared with the NT group. At 1 and 3 h after insult, ScO_2_ values were lower in the TH-H_2_ group compared with the TH group (Fig. 7).

### Occurrence of seizures within 24 h after insult

Analysis of aEEG data showed that seizure occurred within 24 h after insult in all 7 piglets (100%) in the NT group, in 4 of 6 piglets (66.7%) in the TH group, in 2 of 6 piglets (33.3%) in the TH-H_2_ group, and in 6 of 7 piglets (85.7%) in the H_2_ group.

## Discussion

This is the first study in a neonatal HIE piglet model to examine the ability of H_2_ therapy to ameliorate vascular leakage via an analysis of albumin immunohistochemistry. Our findings provide conflicting evidence as to whether H_2_ therapy has potential in alleviating albumin leakage.

In HIE piglet models, HI insult severity is crucial for assessing the efficacy of new therapeutic approaches. Even though few studies have directly used the total duration of LAEEG as an index of HI insult severity, it is associated with biomarkers that determine brain injury severity. In previous work, the total duration of LAEEG during the HI insult was correlated with the cortical/WM injury^31^. In addition, one piglet with an LAEEG duration less than 20 min did not have any brain damage while piglets with a long duration of LAEEG developed seizures. In another study, the HI protocol involved a reduction in the FiO_2_ by between 3 and 4% and its adjustment to maintain the cerebral function monitor at ≤ 5 µV. The duration of the HI insult, including the 10 min of hypotension, determines the severity of the brain damage. A mild insult was defined as a duration of 20 min while a severe insult was defined as a duration of 30 min. The severity was consistent with the histological brain damage^36^. In our model, the longer the duration of LAEEG after the insult, the larger the increase in CBV, which indicated severe brain injury^37^. Based on the literature, we considered that a total duration of LAEEG between 20 and 60 min would indicate moderate-to-severe severity in our model.

In our piglet model, H_2_ did not significantly reduce the albumin extravasation in response to the moderate-to-severe HI insults, as determined by the ratio of albumin stained to unstained areas, even though histological images showed signs of improvement.

Regarding vascular leakage, several mechanisms can be considered. The BBB is a unique physiological barrier formed by the endothelium of cerebral microvessels in which tight junctions regulate the transport of substances between blood and the central nervous system. The BBB is responsible for protecting the brain from neurotoxic substances and delivering essential nutrients^38–40^. The BBB also participates in neural signaling, innate immune responses, and cellular repair and seeks to preserve optimal brain function. Normal BBB function is maintained by neurovascular units comprising vascular cells (endothelial cells, pericytes, and smooth muscle cells), glial cells (astrocytes and microglia), and neurons^41^. When the brain is exposed to certain insults, including HI insults, their impact on the BBB leads to disruption of the above-mentioned critical functions. Because of the complex interactions involved, better understanding of the mechanisms and cells helping to maintain BBB structure and function can also be key to a new therapeutic approach in neonatal HIE research.

HI damage is associated with inflammation and oxidative stress, which have impacts on BBB permeability and angiogenesis. Angiogenesis is essential for the replacement of old vessels and the formation of new immature vessels, which eventually develop into mature and stable vasculature to maintain permeability^12^. Increased BBB permeability allows the infiltration of peripheral lymphocytes and macrophages, which augment the inflammatory response and cause further damage, leading to vasogenic edema^42,43^. Various biomarkers have been combined to detect increased BBB permeability, such as dyes and radiolabeled substances and the immunohistochemical detection of plasma proteins, including albumin^44^. In general, a BBB breach can be assessed by using albumin immunohistochemistry. Being an endogenous plasma protein, albumin avoids the nonphysiological conditions of dyes. Although there is no single and ideal biomarker for the assessment of BBB integrity, more than 500 publications have used albumin as a marker of BBB permeability^44^.

In translational neonatal HIE models, the BBB is damaged after an HI injury, which results in the extravasation of large endogenous plasma proteins such as albumin and smaller injected molecules^45^. In neonatal mice exposed to an HI insult, increased BBB permeability is associated with elevated albumin extravasation, which increases with time after the HI insult. In the same study, infarct area and albumin extravasation were correlated, showing that BBB damage is associated with neuropathological damage^39^. In fetal HIE models, chronic hypoxia reduces pericytes and astrocytic end-feet associated with the increased extravasation of albumin^46^. In clinical HIE, an elevated cerebrospinal fluid/blood albumin ratio (BBB permeability) is observed and a significant correlation is also seen between BBB permeability and free radical injury markers^14^.

Numerous studies have examined the neuroprotective effects of H_2_. Its anti-oxidative, anti-inflammatory, and anti-apoptotic properties are interconnected in a complex manner. Therefore, we focus here on some important studies that emphasize how H_2_ protects the BBB as a speculation. One possible mechanism involves modulation of nuclear factor erythroid 2-related factor (NRF2), which is the key transcription factor protecting cells against reactive oxygen species, pro-inflammatory stimuli, and subsequent apoptosis. In the BBB, NRF2 promotes the expression of tight junction proteins, maintains mitochondrial functions, and enhances ATP production. Activation of NRF2 is associated with protection of the BBB integrity and improvements in cognition^47,48^. In septic mice, inhalation of 2% H_2_ alleviates BBB damage, decreases pro-inflammatory cytokines, increases anti-inflammatory factors, and improves survival by enhancing NRF2-dependent downstream signaling pathways^49^. In another study, BBB disruption and brain edema were improved by 2% H_2_ via increased NRF2 expression^19^.

Here, we examined the patterns of albumin-reactive areas (Fig. 4). In general, at a low-power magnification, the signs of albumin leakage were more common in the central areas, likely around the choroid plexus, compared with the periphery. In perinatal HI insult, the choroid plexus shows extensive necrosis after the insult^50^. Neutrophils contribute to the brain swelling in perinatal HI brain injury and most neutrophils are located in the choroid plexus^51^. The choroid plexus produces cerebrospinal fluid, plays a role in secretory and immune function, and transports nutrients and metabolites across the barrier^52^. Even though we focused on the consequences of vascular leakage in the BBB in this study, understanding of the alterations in permeability across the choroid plexus of the blood–cerebrospinal fluid barrier should also be useful for studying pathogenesis and disease progress.

In terms of cerebral hemodynamics, cerebral blood volume increased from 12 to 24 h after insult in the H_2_ and H_2-_TH groups, suggesting that H_2_ may exert an effect, whether alone or combined with TH, that could aid in the neuronal survival by promoting greater blood flow to the neurons, and increased consumption of oxygen might be reflected in the form of lower ScO_2_^53^.

Regarding the occurrence of seizure, most of the piglets in the H_2_-TH group did not develop seizures within 24 h after insult. By contrast, most of the piglets that received H_2_ ventilation alone had seizures within 24 h. However, in this study, albumin leakage was assessed at day 5 after insult, meaning that the relationship between seizure occurrence within 24 h and vascular leakage at day 5 is difficult to interpret.

In our study, H_2_ therapy (both alone and combined with TH) did not effectively ameliorate vascular leakage. The interpretation of this finding should consider multiple factors, such as target cells or area, severity of insult, and whether or not the mechanisms of action of the therapies overlap with each other at particular phases of HIE. First, this study assessed vascular leakage. The histological outcomes of the neurons and the supporting neural cells were not investigated. Previously, by using the same piglet model, our group investigated the histological outcomes of acute renal injury after TH treatment and found that renal fibrosis was not improved with TH^54^. Because the kidneys are highly vascularized, we speculated that the reduction in renal blood flow with both asphyxia and TH are the main reasons why TH did not ameliorate the renal fibrosis.

Next, our previous study of H_2_ concluded that the 5-day neurological outcome was better with combined therapy than with TH alone^20^. In that study, the insult severity was highly variable, from mild to severe, whereas the current study focused on a moderate-to-severe insult.

Regarding combined H_2_ and TH therapy, Kovács et al. concluded that neither H_2_ nor CO_2_ combined with TH showed superior neuroprotective effects in their piglet model. The authors mentioned the possibility of their combination resulting in neutral or even harmful effects^35^. Similar outcomes were observed when erythropoietin was combined with TH, with the High-dose Erythropoietin for Asphyxia and Encephalopathy (HEAL) study concluding that there was no significant reduction in death or neurodevelopmental impairment at 2–3 years of age and that the treatment was associated with serious adverse events in infants with moderate-to-severe HIE^55^. In preclinical studies, erythropoietin had good outcomes when used alone but mixed outcomes when combined with TH due to the activation of similar neuroprotective pathways during the acute phase^56–59^. Similarly, in the present study, H_2_ might reverse the improvement in vascular leakage induced by TH, at least based on the previous findings.

There are several limitations to this study. First, we focused on a moderate-to-severe HI insult. Therefore, the effectiveness of H_2_ in mild and very severe insults is unknown. In this study, the histological damage to cerebral vasculatures (e.g., the condition of endothelial cells) was not examined, even though our main focus was on albumin extravasation. The next step is to determine the optimal timing for starting therapy after HI and the duration of H_2_ therapy in order to evaluate whether a single or combined approach is effective. Finally, anti-oxidative, anti-inflammatory, and anti-apoptotic biomarkers were not studied, meaning that the factor underlying the improvement in albumin leakage was not identified. Nonetheless, this translational study revealed the neuroprotective potential of H_2_ therapy for neonatal HIE.

To conclude, our study could not prove the efficacy of H_2_ ventilation alone or combined with TH for ameliorating vascular leakage. However, H_2_ ventilation has potential neuroprotective effects that are worth exploring. As a physiological gas that is potentially harmless in vivo, H_2_ may be a suitable candidate for clinical use in the developing and vulnerable brain. H_2_ ventilation is also a feasible method compared with H_2_ saline in newborns because fluid management can be challenging in HIE. Overall, the benefits of H_2_ appear to outweigh the risks. Future studies should focus on a carefully designed translational model with a specific insult severity, target cells (e.g., neurons, astrocytes, and BBB endothelial cells), and an appropriate set of biomarkers to better understand and highlight the effectiveness of H_2_.

## Materials and methods

### Ethical approval and animal preparation

The study protocol was approved by the Animal Care and Use Committee for Kagawa University (15070-1) and in accordance with Animal Research: Reporting In Vivo Experiments guidelines. All methods were carried out in accordance with relevant guidelines and regulations. Thirty-three newborn piglets within 24 h after birth (21 males, 12 females; body weight ranging from 1450 to 2150 g) were anesthetized and surgically prepared.

Because the experimental procedures are described in detail in previous articles, the procedures are only briefly noted in this report^20,21^. The piglets were placed under a radiant warmer and their activities and alertness were briefly observed. Anesthesia was induced with 1–2% isoflurane (Forane^®^ inhalant liquid; Abbott Co., Tokyo, Japan) in air using a facemask. Each piglet was then intubated and mechanically ventilated. An umbilical vein catheter was inserted for blood pressure monitoring, as well as an umbilical artery catheter for blood sampling. After cannulation, the piglets were anesthetized with fentanyl citrate at an initial dose of 10 µg/kg followed by continuous infusion at 5 µg/kg/h and were paralyzed with pancuronium bromide at an initial dose of 100 µg/kg followed by continuous infusion at 100 µg/kg/h. Maintenance solution was infused continuously at a rate of 4 mL/kg/h via the umbilical vein. Arterial blood samples were taken at critical points and when clinically indicated throughout the experiment. Each piglet was then placed in a copper mesh-shielded cage under a radiant warmer to maintain a RT of 38.0 °C ± 0.5 °C. Inspired gas was prepared by mixing O_2_ and N_2_ gases to obtain the oxygen concentrations required for the experiment. Ventilation was adjusted to maintain PaO_2_ and PaCO_2_ within their normal ranges. MABP was measured and recorded via the umbilical arterial catheter.

### Time-resolved near-infrared spectroscopy and analysis

A portable three-wavelength TRS system (TRS-10; Hamamatsu Photonics K.K., Hamamatsu, Japan) was applied using probes attached to the head of each piglet. The light emitter and detector optodes were positioned on the parietal region of each piglet with a 30-mm interoptode distance. In the TRS system, a time-correlated single-photon-counting technique is used for detection. The concentrations of oxyhemoglobin (oxyHb) and deoxyhemoglobin (deoxyHb) are calculated from the absorption coefficients of oxyHb and deoxyHb, with the assumption that background absorption is due only to 85% (by volume) water. The total cerebral Hb concentration (totalHb), ScO_2_, and CBV were calculated as described previously^60,61^.

### Amplitude-integrated electroencephalography

Neural activity was measured by aEEG (Nicolet One; Cardinal Health, Inc., Dublin, OH). All electrical devices and the copper mesh shield were grounded. The signal was displayed on a semi-logarithmic scale at a low speed (6 cm/h). Measurements were conducted every second. Gold-plated electrode needles were placed at the P3 and P4 positions, which corresponded to the left and right parietal regions of the head. A maximum amplitude < 5 µV was defined as LAEEG. The aEEG data were examined for the occurrence of seizure within 24 h after insult.

### Hypoxic-ischemic insult protocol

Because the details were reported in our previous work^21^, only an outline of the HI insult protocol is provided here (Fig. 1). Hypoxia was induced by a reduction in the inspired oxygen concentration of the ventilator to 4% after at least 120 min of stabilization from the initial anesthetic induction. To obtain an LAEEG pattern (< 5 µV), the inspired oxygen concentration was further reduced, with adjustments to avoid causing cardiopulmonary arrest. From the beginning of LAEEG, the insult was continued for 30 min. FiO_2_ was decreased (1% decrements) or increased (1% increments) during the insult to maintain the LAEEG, HR (> 130 beats/min), and MABP (> 70% of baseline). LAEEG was maintained for 20 min. For the final 10 min of the 30-min insult, if the MABP exceeded 70% of the baseline, hypotension was induced by decreasing the FiO_2_. Resuscitation was performed when the CBV value dropped below 30% and/or the MABP declined below 70% of baseline. Hypoxia was terminated by resuscitation with 100% oxygen. NaHCO_3_ was used to correct a base deficit (base excess below − 5.0 mEq/L) to maintain a pH of 7.3–7.5. After 10 min of 100% FiO_2_, the ventilator rate and FiO_2_ were gradually reduced to maintain an SpO_2_ of 95–98%.

### Post-insult treatment

After the HI insult, 33 piglets were randomized into four groups: HI insult with normothermia (NT, n = 9), HI insult with H_2_ ventilation (H_2_, n = 8), HI insult with TH (TH, 33.5 °C ± 0.5 °C, n = 8), and HI insult with H_2_ ventilation with TH (H_2_-TH, 2.1–2.7% H_2_, n = 8). Whole-body hypothermia was achieved using a cooling blanket (Medicool; MAC8 Inc., Tokyo, Japan) after resuscitation. The piglets were cooled to 33.5 °C ± 0.5 °C for 24 h and then rewarmed at 1 °C/h using a blanket. RT was used as the measure of body temperature. The temperature of the incubator was maintained at 28–32 °C. Once the piglets were weaned off the anesthesia and ventilator and extubated, they were allowed to recover and were maintained for 5 days in the incubator. Piglets were fed 50–100 mL artificial animal milk via a nasogastric tube every 6 h. The presence of seizures was recognized clinically as rhythmic pathologic movements (cycling) and tonic postures sustained between cycling episodes. If seizures occurred, the piglet was treated with phenobarbital (20 mg/kg) via intramuscular injection. If seizures persisted, the piglet was treated with two successive anticonvulsant doses. If seizures persisted after two successive anticonvulsant doses, the piglet was euthanized.

### Hydrogen therapy

For H_2_ ventilation, two types of cylinders were used: one contained a gas mixture comprising 3.8% H_2_ and 96.2% N_2_; the other contained 100% O_2_. The H_2_ concentration depended on the oxygen requirement of each piglet. Therefore, the H_2_ concentration was usually between 2.1 and 2.7 (FiO_2_ range, 0.21–0.4) during the therapy. H_2_ was delivered through the ventilator for 24 h. The concentration of H_2_ was measured by a portable gas monitor (GX-8000; RIKEN KEIKI Co., Ltd., Japan). After 24 h of treatment, the hydrogen–nitrogen gas mixture was replaced with the air compressor.

For piglets given TH, their temperature was automatically controlled to maintain the target temperature (RT, 33–34 °C) during TH and rewarmed at a rate of 1 °C/h by a cooling blanket. Anesthesia was stopped at the beginning of the rewarming period. For NT piglets, the RT was monitored continuously to maintain a normal range (38–39 °C) under the radiant warmer under anesthesia-ventilation for 24 h after the insult. The anesthesia was then stopped, which was followed by extubation.

### Histological assessment

For the euthanization of piglets on day 5 after the insult, their face was inserted into a mask and inhalation anesthesia was administered. The anesthetic agent isoflurane was introduced via a vaporizer. The vapor was inhaled until respiration ceased and death ensued. The brain of each piglet was perfused with 0.9% saline and 4% phosphate-buffered paraformaldehyde via cannulation of the left ventricle and an incision into the right atrial auricle. Brain tissue was histologically evaluated, and irregularities were graded according to a histopathology grading scale for a piglet model of posthypoxic encephalopathy, which has also been validated^31^. Coronal blocks of the GM, WM, hippocampus, and cerebellum were embedded in paraffin and cut with a microtome at 4 μm.

Albumin immunohistochemistry was performed using goat polyclonal anti-pig albumin antibody (1:200, Cat A100-110P; Bethyl Laboratories, Inc., Montgomery, TX), as instructed by the manufacturer’s protocol. Whole areas in sections were analyzed, and albumin-stained areas were identified using ImageJ software (National Institutes of Health, Bethesda, MD). In our model, albumin leakage was mainly seen in the subcortical structures, especially the basal ganglia and periWM, and superficial cortical GM and subWM. The ratio of albumin stained to unstained areas was analyzed and compared among the four groups.

### Neurological assessment

Soon after the piglets were nursed in the incubator, neurological function was observed by examiners who were blinded to the protocols. Neurological examination was conducted every 6 h for 5 days from day 1 to day 5 post-insult. The neurological scoring comprised nine neurological items: a, respiration; b, consciousness; c, orientation; d, ability to walk; e, ability to control the forelimbs; f, ability to control the hind limbs; g, maintenance of tone; and h, pathological movements (scored as: 2, normal; 1, moderately abnormal; or 0, definitely pathological). The minimum score was 0 and the maximum, indicating a normal healthy piglet, was 18^31^.

### Data analysis

GraphPad Prism 9 (GraphPad Software, La Jolla, CA) was used for all statistical analyses. To demonstrate the efficacy of H_2_ under moderate-to-severe insult conditions, any of the 33 piglets with a total duration of LAEEG below 20 min and above 60 min were excluded from the analysis (2 from the NT group, 1 from the H_2_ group, 2 from the TH group, and 2 from the H_2_-TH group). The final total number of piglets was 26 (NT = 7, H_2_ = 7, TH = 6, and H_2_-TH = 6). Values are expressed as the mean (standard deviation [SD]) for physiological and blood gas data. For the duration of LAEEG, mean (SEM) was used. Physiological data and blood gas data were compared among the four groups at each time point (baseline and 0, 1, 6, 12, and 24 h after the insult). For the comparison of each time point with the baseline value, Dunnett’s multiple comparisons test was used. TRS and ScO_2_ values are expressed as the mean (SD). To compare the ratio of albumin stained to unstained areas among the groups, the Steel–Dwass test was performed and the values were expressed as the median (interquartile range). For the NS from day 1 to day 5 among all groups, one-way ANOVA followed by Tukey’s multiple comparison test was used. Values are expressed as the median (interquartile range). A p value < 0.05 was considered statistically significant.

## Supplementary Information

Supplementary Figure S1.

Supplementary Figure S2.

Supplementary Figure S3.

Supplementary Figure S4.

Supplementary Information.

## Data Availability

The datasets generated during and/or analyzed during the current study are available from the corresponding author on reasonable request.
