# Supplementary figures and images for: Conflicting findings on the effectiveness of hydrogen therapy for ameliorating vascular leakage in a 5-day post hypoxic-ischemic survival piglet model
Source: Sci Rep. 2023 Jun 28;13:10486. doi: 10.1038/s41598-023-37577-0 (PMC10307881; doi:10.1038/s41598-023-37577-0)

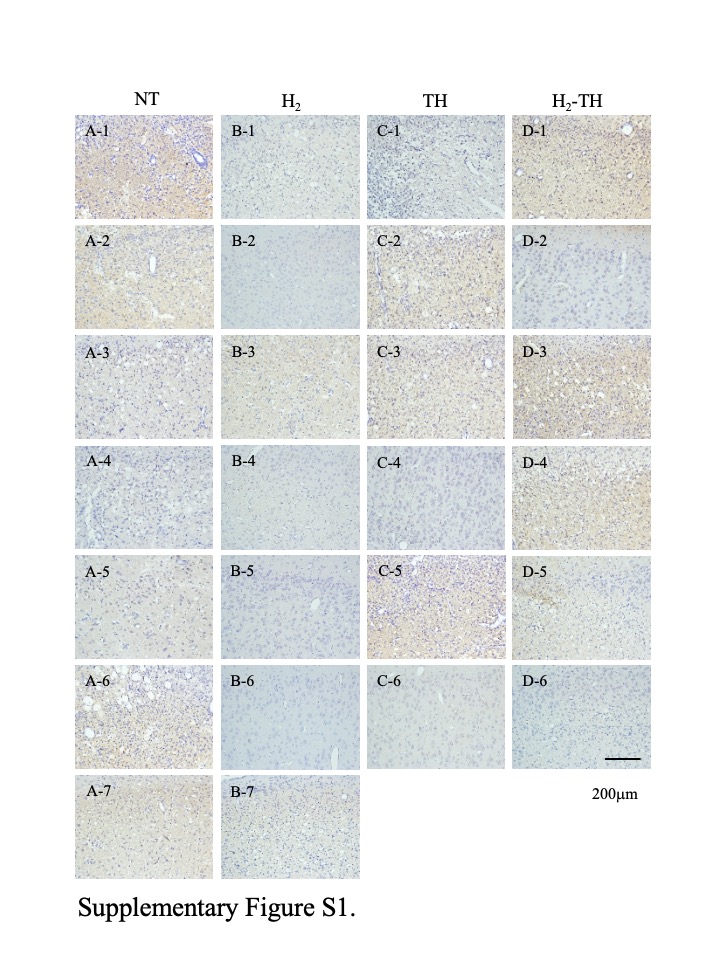

Supplement: Supplementary file 1 — Supplementary Figure S1. [file 41598_2023_37577_MOESM1_ESM.jpg]

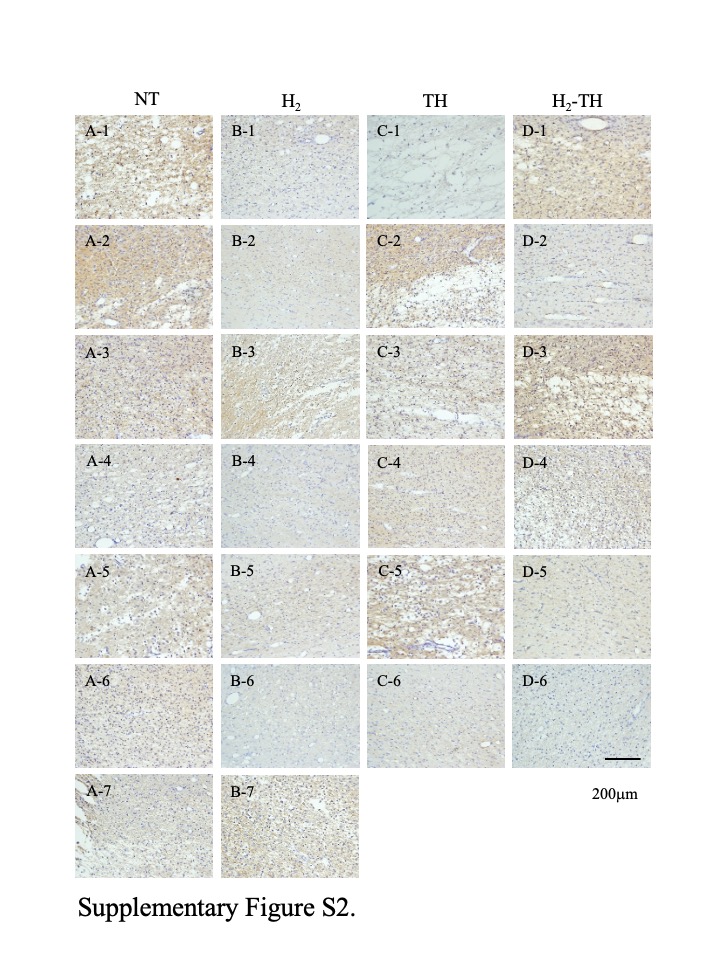

Supplement: Supplementary file 2 — Supplementary Figure S2. [file 41598_2023_37577_MOESM2_ESM.jpg]

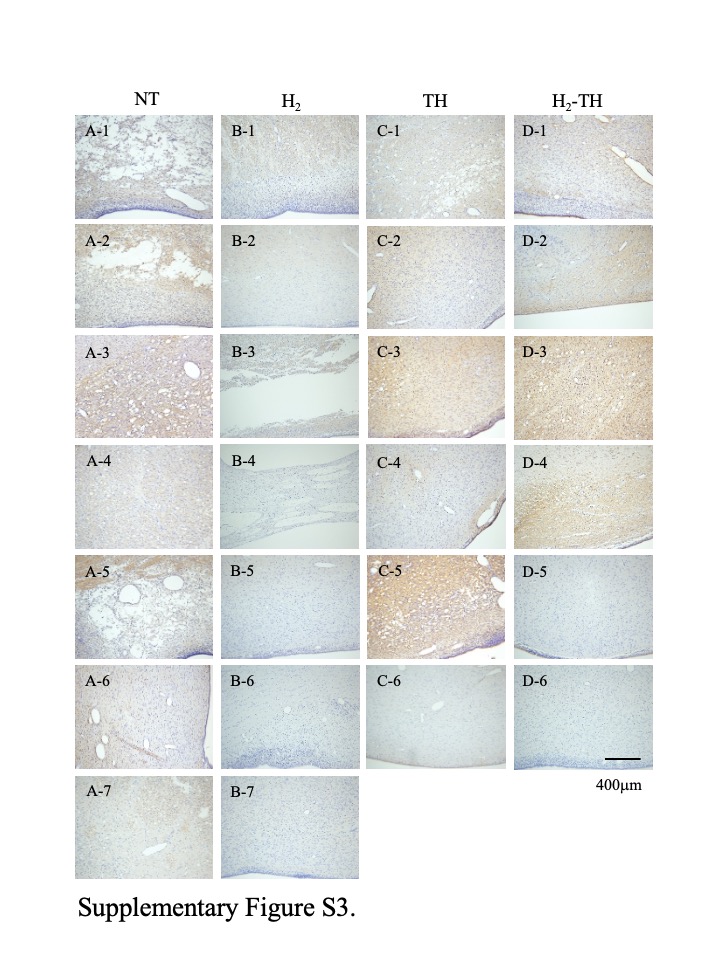

Supplement: Supplementary file 3 — Supplementary Figure S3. [file 41598_2023_37577_MOESM3_ESM.jpg]

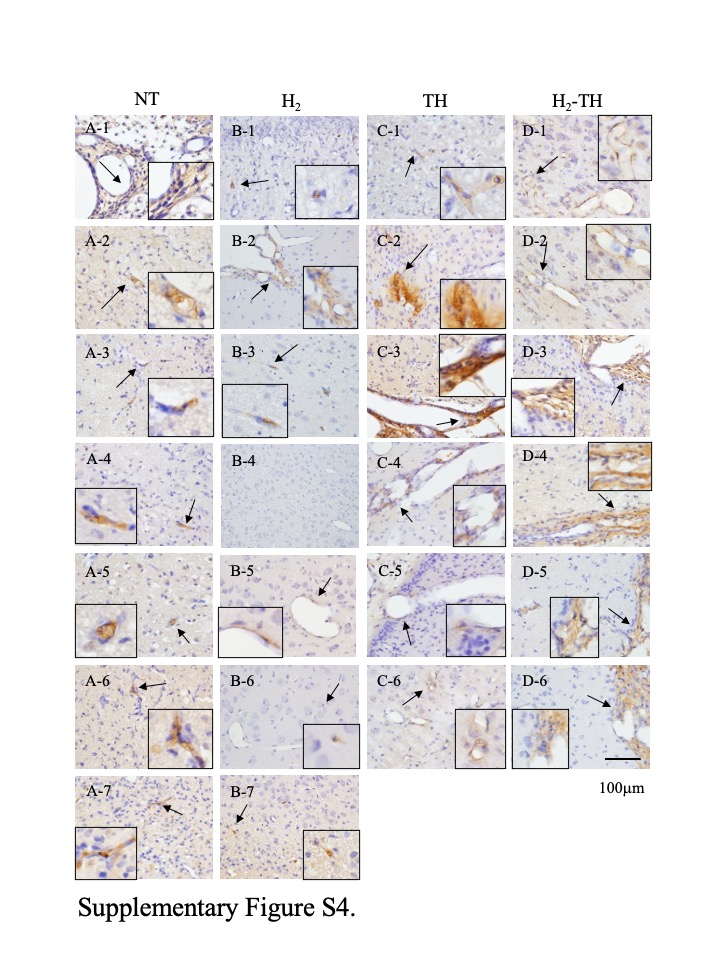

Supplement: Supplementary file 4 — Supplementary Figure S4. [file 41598_2023_37577_MOESM4_ESM.jpg]
