# Supplementary material for: Conflicting findings on the effectiveness of hydrogen therapy for ameliorating vascular leakage in a 5-day post hypoxic-ischemic survival piglet model
Source: Sci Rep. 2023 Jun 28;13:10486. doi: 10.1038/s41598-023-37577-0 (PMC10307881; doi:10.1038/s41598-023-37577-0)
Supplement: Supplementary file 5 — Supplementary Information. [file 41598_2023_37577_MOESM5_ESM.docx]

**Supplementary information**

**Article in *Scientific Reports***

**Conflicting findings on the effectiveness of hydrogen therapy for ameliorating vascular leakage in a 5-day post hypoxic-ischemic survival piglet model**

Authors and affiliation

Yinmon Htun^1^, Shinji Nakamura^1^, Yasuhiro Nakao^1^, Tsutomu Mitsuie^2^, Kenichi Ohta^3^, Makoto Arioka^4^ Takayuki Yokota^1^, Eri Inoue^1^, Kota Inoue^1^, Toi Tsuchiya^1^, Kosuke Koyano^4^, Yukihiko Konishi^1^, Takanori Miki^3^, Masaki Ueno^5^, and Takashi Kusaka^1*^

^1^Department of Pediatrics, Faculty of Medicine, Kagawa University, 1750-1 Mikicho, Kitagun, Kagawa 761-0793, Japan

^2^Medical Engineering Equipment Management Center, Kagawa University Hospital, Kagawa University, 1750-1 Mikicho, Kitagun, Kagawa 761-0793, Japan

^3^Department of Anatomy and Neurobiology, Faculty of Medicine, Kagawa University, 1750-1 Mikicho, Kitagun, Kagawa 761-0793, Japan

^4^Maternal Perinatal Center, Kagawa University Hospital, Kagawa University, 1750-1 Mikicho, Kitagun, Kagawa 761-0793, Japan

^5^Department of Pathology and Host Defense, Faculty of Medicine, Kagawa University, 1750-1 Mikicho, Kitagun, Kagawa 761-0793, Japan

Method

Same as Albumin Immunohistochemistry in the manuscript

Results

Immunohistochemical images of albumin in the GM, subWM, and subcortical structures in all piglets of the four groups are shown in Supplemental Figures S1–S4, respectively. In the NT group, moderate-to-strong immunohistochemical staining was observed in GM and subWM areas, with clear tissue destruction in the deep structures in five cases (including three with severe destruction) and mild destruction in one case. In the H_2_ group, mild-to-moderate immunohistochemical staining was observed in GM and subWM areas, with clear tissue destruction in the deep structures in two cases (including one with severe destruction) and mild destruction in one case. In the TH group, mild-to-strong immunohistochemical staining was observed in GM and subWM areas, with clear tissue destruction in the deep structures in two cases and mild destruction in one case. In the H_2_-TH group, mild-to-strong immunohistochemical staining was observed in GM and subWM areas, with clear tissue destruction in the deep structure in two cases and mild destruction in one case. Regarding the subcortical structures, strong immunohistochemical staining was observed in the NT group. In the TH group, two cases showed strong staining. The H_2_-TH group showed a similar tendency to the TH group. However, in the H_2_ group, the staining was relatively weak compared with the other groups. All representative images of albumin immunostaining used in the immunohistochemical analysis are shown in Supplementary Figures S1, S2, S3, and S4.

Supplementary figure legends

Supplementary Figure S1.

Representative images of albumin immunostaining in the GM in the NT (A-1, -2, -3, -4, -5, -6, and -7), H_2_ (B-1, -2, -3, -4, -5, -6, and -7), TH (C-1, -2, -3, -4, -5, and -6), and H_2_-TH (D-1, -2, -3, -4, -5, and -6) groups. Scale bar, 200 μm.

Supplementary Figure S2.

Representative images of albumin immunostaining in the subWM in the NT (A-1, -2, -3, -4, -5, -6, and -7), H_2_ (B-1, -2, -3, -4, -5, -6, and -7), TH (C-1, -2, -3, -4, -5, and -6), and H_2_-TH (D-1, -2, -3, -4, -5, and -6) groups. Scale bar, 200 μm.

Supplementary Figure S3.

Representative images of albumin immunostaining in the subcortical structures in the NT (A-1, -2, -3, -4, -5, -6, and -7), H_2_ (B-1, -2, -3, -4, -5, -6, and -7), TH (C-1, -2, -3, -4, -5, and -6), and H_2_-TH (D-1, -2, -3, -4, -5, and -6) groups. Scale bar, 400 μm.

Supplementary Figure S4.

Representative enlarged images of albumin immunostaining in the GM in the NT (A-1, -2, -3, -4, -5, -6, and -7), H_2_ (B-1, -2, -3, -4, -5, -6, and -7), TH (C-1, -2, -3, -4, -5, and -6), and H_2_-TH (D-1, -2, -3, -4, -5, and -6) groups to show vascular leakage of albumin. Arrows indicate vessels exhibiting increased vascular permeability. Scale bar, 100 μm.
